# 

**Published:** 1878-06

**Authors:** 


### Contents of June Number.

### ORIGINAL.

ART. I.—Purpura, Occurring in Nervous Diseases. By J. B. Andrews, A. M., M. D. .... 401
ART. III.—The Obstetrical Forceps. By Prof. James P. White, M. D... 408
ART. III.—Placenta Prævia. Its Treatment. By Charles F. A. Nichell, M. D .... 415
ART. IV.—Abstract of the Proceedings of the Buffalo Medical Association, May 7th, 1878, .... 419

### CORRESPONDENCE.

A New Speculum. By Dr. S. G. Dorr, .... 423

### MISCELLANEOUS.

Prevalence of Malaria, 424. Intra-Venous Injection of Milk, 426. Injuries of the Head, 427. Post-Partum Hæmorrhage treated by injection of Hot Water, 428. Foreign Bodies in the Ear, 428. The Excision of Hard Chancres, 429. The Temptations of Physicians, 429. On the Treatment of Acne, 430. Poisoning by Chlorate of Potash, 431. The Indications for Drainage of the Knee-Joint, 432. Limitations of the Senses, 432. Freckles, 432.

### EDITORIAL.

American Medical Association, .... 433
—Directory of the Places of Meeting .... 433
—Programme of the First General Session .... 433
—Fare to Buffalo .... 434
Medical Notes and Comments .... 434
Business Notices .... 436
Popular Science Monthly .... 436
Lactopeptine .... 437

BOOKS REVIEWED:
Practical Gynecology. Smith .... 437
Artificial Anæsthesia. Turnbull .... 438
Landmarks, Medical and Surgical. Holden .... 438
Transactions of the Medical Society of the State of New York.. 439
Element of Therapeutics .... 439
Vest Pocket Anatomist, .... 440

Books and Pamphlets Received .... 440

THE BUFFALO

